# Synthesis, Biological Evaluation and Docking Studies of 13-Epimeric 10-fluoro- and 10-Chloroestra-1,4-dien-3-ones as Potential Aromatase Inhibitors

**DOI:** 10.3390/molecules24091783

**Published:** 2019-05-08

**Authors:** Rebeka Jójárt, Péter Traj, Édua Kovács, Ágnes Horváth, Gyula Schneider, Mihály Szécsi, Attila Pál, Gábor Paragi, Erzsébet Mernyák

**Affiliations:** 1 Department of Organic Chemistry, University of Szeged, Dóm tér 8, H-6720 Szeged, Hungary; j.rebeka05@gmail.com (R.J.); trajpeter@gmail.com (P.T.); eduakovacs91@gmail.com (É.K.); horvathagnesttik@gmail.com (Á.H.); schneider@chem.u-szeged.hu (G.S.); 2 1st Department of Medicine, University of Szeged, Korányi fasor 8–10, H-6720 Szeged, Hungary; szecsi.mihaly@med.u-szeged.hu; 3 Department of Medicinal Chemistry, University of Szeged, Dóm tér 8, H-6720 Szeged, Hungary; palattila95@gmail.com; 4 MTA-SZTE Biomimetic Systems Research Group, University of Szeged, Dóm tér 8, H-6720 Szeged, Hungary; paragi@sol.cc.u-szeged.hu; 5 Institute of Physics, University of Pecs, Ifjúság útja 6, H-7624 Pécs, Hungary

**Keywords:** 13α-estrone, Selectfluor, aromatase, docking, TEMPO, single electron transfer

## Abstract

Fluorination of 13-epimeric estrones and their 17-deoxy counterparts was performed with Selectfluor as the reagent. In acetonitrile or trifluoroacetic acid (TFA), 10β-fluoroestra-1,4-dien-3-ones were formed exclusively. Mechanistic investigations suggest that fluorinations occurred via SET in acetonitrile, but another mechanism was operative in TFA. Simultaneous application of N-chlorosuccinimide (NCS) and Selectfluor in TFA led to a 1.3:1 mixture of 10β-fluoroestra-1,4-dien-3-one and 10β-chloroestra-1,4-dien-3-one as the main products. The potential inhibitory action of the 10-fluoro- or 10-chloroestra-1,4-dien-3-one products on human aromatase was investigated via in vitro radiosubstrate incubation. The classical estrane conformation with *trans* ring anellations and a 13β-methyl group seems to be crucial for the inhibition of the enzyme, while test compounds bearing the 13β-methyl group exclusively displayed potent inhibitory action with submicromolar or micromolar IC_50_ values. Concerning molecular level explanation of biological activity or inactivity, computational simulations were performed. Docking studies reinforced that besides the well-known Met374 H-bond connection, the stereocenter in the 13 position has an important role in the binding affinity. The configuration inversion at C-13 results in weaker binding of 13α-estrone derivatives to the aromatase enzyme.

## 1. Introduction

Aromatase is responsible for the aromatization of androgens to estrogens [1]. The overproduction of estrogens stimulates the proliferation of estrogen-sensitive cells, leading to estrogen-dependent cancers. The proliferative action of estrogens might be prevented by inhibition of the aromatase enzyme [2]. Aromatase inhibitors can be categorized by their mechanism of action. Type I inhibitors are known as steroidal, and type II as nonsteroidal inhibitors [1]. The type I compounds are usually related to substrates of the enzyme and they are either competitive inhibitors or act as suicide inhibitors [3,4]. Formestane and Atamestane belong to the type I group. Formestane is considered to be the structural analog of androstenedione and it was the first member to enter clinical trials. Despite their high inhibitory potency, both agents have unfavorable metabolism and poor oral bioavailability. These disadvantages led to the discovery of a more potent compound, named Exemestane, acting both as a competitive and irreversible inhibitor. Additionally, it is efficient in metastatic breast cancer after the failure of selective estrogen receptor modulators (SERMs) [3]. This agent has substantial advantages over the nonsteroidal aromatase inhibitors. Thanks to the mentioned benefits of Exemestane, subsequent novel therapeutics were developed using a steroidal backbone as the scaffold. Nowadays, there is a high need for new aromatase inhibitory agents, because drawbacks as androgenic effect or metabolism by other CYP enzymes could still not be avoided.

Attempts have been made in recent decades to decrease the side-effects of the known inhibitors by performing minor structural modifications on the sterane skeleton [1]. Certain modifications were based on retaining the ring A dienone and androstane structure. One of the most explored groups of aromatase inhibitors is the 19-substituted androstane class. Marcotte and Robinson published work on C-19-modified androsta-4-en-3-one derivatives [5]. The 19-monofluoro and its difluoro counterpart displayed aromatase inhibitory activity with a Ki value of 1 µM. These literature data indicate that both the presence of an enone moiety in ring A and the fluorinated β-oriented angular 10-methyl group are advantageous structural elements concerning aromatase inhibition. Halogenation was also performed on the androsta-1,4-diene-3,17-dione skeleton [6]. Chlorine was introduced onto C-19, and the resulting compound displayed potent inhibitory activity with a 1-micromolar IC_50_ value. Not only ring A, but also ring D modifications have been performed. Sherwin et al. published the comparison of the inhibitory data of 17-oxo and the parent 17-deoxy androsta-1,4-dien-3-one compounds [7]. They concluded that the removal of the 17-oxo function causes only minor differences in the aromatase inhibitory potential of androsta-1,4-dien-3-ones.

**Scheme 1:**
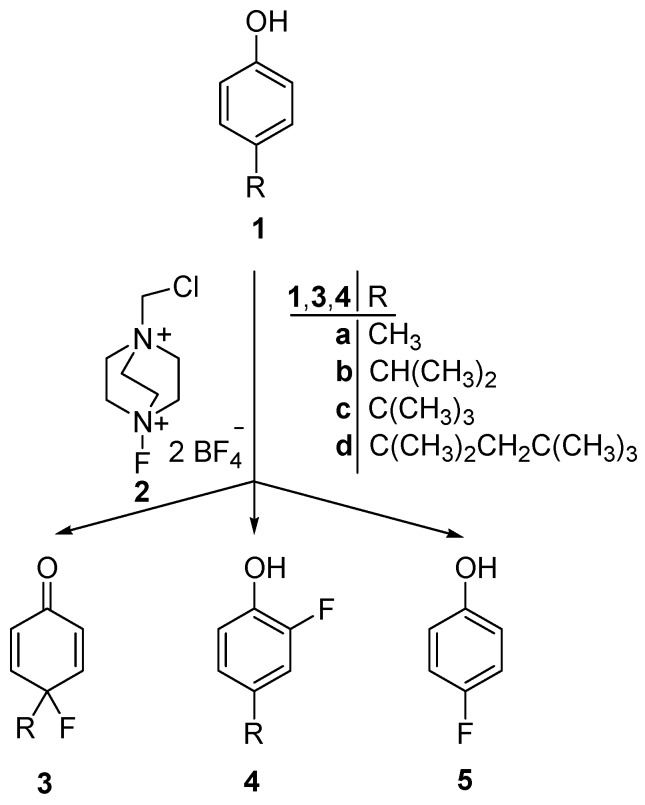
Fluorination of *p*-substituted phenols (**1**) with Selectfluor (**2**).

Incorporation of fluorine into a biomolecule may lead to beneficial biological properties [8]. The C–F bond participates in attractive interactions with hydrogen bond donors, certain polar functional groups, and hydrophobic moieties. This is due to the large C–F bond polarization, which originates from the high electronegativity of fluorine. Fluorinated molecules usually have high binding affinity to certain proteins and increased metabolic stability. Recently, a class of stable and crystalline N–F fluorinating agents has been developed. 1-Chloromethyl-4-fluoro-1,4-diazoniabicyclo[2.2.2]octane bis(tetrafluoroborate) (Selectfluor (**2**), F-TEDA-BF_4_) belongs to the latter group and behaves as a selective fluorinating agent with high functional group tolerance [9,10,11,12]. Selectfluor is exceptionally stable and may serve as a fluoronium cation source. It is soluble in a few polar solvents, namely, acetonitrile, *N*,*N*-dimethylformamide (DMF), nitromethane and water. Literature data reveal that the type of fluorination of aromatic molecules with Selectfluor strongly depends on both the nature and the position of the aromatic ring substituents as well as on reaction conditions. Pravst et al. performed fluorinations of *p*-substituted phenols (**1**) using Selectfluor (**2**) as fluorinating agent in acetonitrile or methanol under different conditions (Scheme 1) [13]. The greatest solvent-dependent difference in product distributions was observed starting from 4-methylphenol (**1a**), where products **3a** and **4a** were formed in a ratio of nearly 2:1 in acetonitrile (reflux, 2 h), but in **3a**:**4a** = 0:1 in methanol (reflux, 2 h). Concerning the largest substituent in the starting compound (**1d**), no dienone (**3d**) was formed, and the **4d**:**5d** = 88:12 ratio was the same, independent of the solvent used.

**Scheme 2:**
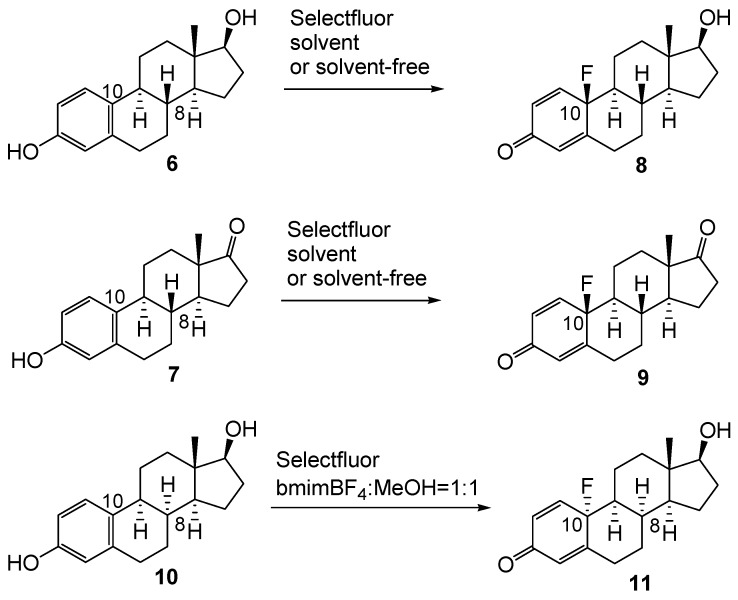
Fluorination of estrone derivatives (**6**, **7** or **10**) with Selectfluor (**2**).

According to the literature, 17β-estradiol (**6**), estrone (**7**) or their certain ring D-substituted derivatives can be converted to 10β-fluoroestra-1,4-dien-3-one analogues (**8** and **9**) with Selectfluor in different solvents (acetonitrile or bmimBF_4_:MeOH = 1:1, H_2_O) or under solvent-free conditions (Scheme 2) [14,15,16,17]. Independent of the nature of the solvent, all reactions resulted in dienones **8** and **9** as main products. *ortho*-substituted derivatives were observed only in trace amounts. Bogautdinov et al. reported the stereoselective fluorination of the 8α-epimer of 17β-estradiol (**10**) in the 1:1 mixture of bmimBF_4_ and methanol (Scheme 2) [14]. They proved that the reaction leads to 10-fluoro derivative **11** as the main product with α-orientation of fluorine. It was established that the configuration of C-8 influences the chirality of the newly formed C-10 stereogenic center.

**Figure 1:**
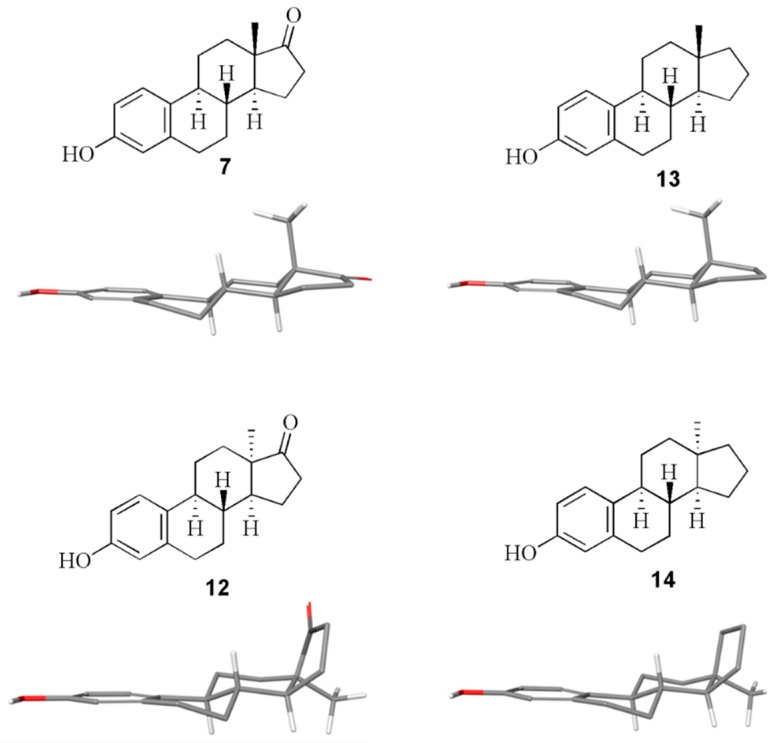
The structure of 13-epimeric estrones (**7** and **12**) and their 17-deoxy counterparts (**13** and **14**).

Natural estrone derivatives (**6** and **7**) exhibit a relatively rigid molecular framework with well-defined distances between the two oxygen functionalities, which might be essential in the binding of the biomolecule to its receptors or enzymes. In contrast to natural 13β-estra-1,3,5(10)-trienes, 13α derivatives possess a *cis*-junction of the C and D rings, a quasi-equatorial 13α-methyl group and a ring D that is directed to the β-side (Figure 1) [18,19]. 13α-Estrone derivatives exist either in a usual conformation (with chair ring C) or in an unusual steroid conformation (with a twist-boat ring C). The conformational changes lead to a complete loss of estrogenic activity in estrone (**7**) or 17α/β-estradiols with inverted configuration at C-13 [20]. Thus, the inversion of certain chiral carbon atoms of the estrane core may lead to completely different biological behavior. Accordingly, 13α-estrone (**12**, Figure 1) may serve as a suitable scaffold for the design of biologically active estrane derivatives lacking estrogenic behavior.

**Scheme 3:**
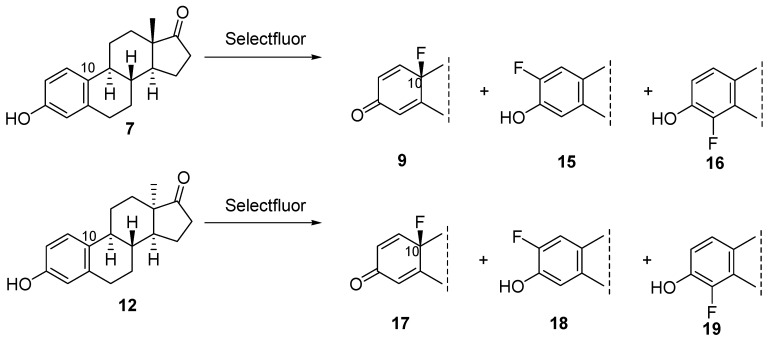
Fluorinations of estrone (**7**) and 13α-estrone (**12**) with Selectfluor (**2**).

Here, we aimed to perform fluorination of hormonally inactive 13α-estrone **12** in order to obtain novel potential steroidal aromatase inhibitors bearing the 1,4-dien-3-one structural moiety in ring A. We aimed to examine the chemo- and stereoselectivity of the fluorination with Selectfluor (**2**) under various conditions. The investigation of the reaction mechanism was planned by adding a radical scavenger to the reaction mixture. Another substrate, 17-deoxy-13α-estrone **14**, was also subjected to these transformations with the aim of investigating the influence of the lack of the 17-oxo group on the scope of the reactions. Comparative studies in the 13β- and 13α-estrone series (from starting compounds **7** and **12**–**14**) have also been planned. The determination of the potential inhibitory action of the 10-fluorinated estra-1,4-dien-3-one products (**9**, **17**, **20**, and **21**; Scheme 3) on human aromatase enzyme was planned via in vitro radiosubstrate incubation. Finally, having obtained a molecular-level insight into the binding properties of the 10-halo-13-epimeric estrane derivatives (**9**, **17**, and **20**–**22**) structure-activity information was collected and computational investigations were performed. These docking simulations helped to understand the more profound consequence of our chemical modifications concerning the ligand–receptor interaction.

## 2. Results and Discussion

### 2.1. Chemistry

Fluorinations of 13-epimeric estrones (**7** and **12**) with Selectfluor (**2**) were performed in different solvents (Scheme 3). Based on literature results, two solvents were selected: acetonitrile and methanol [12]. Reactions were performed at room temperature or at 80 °C. First, chemo- and regioselectivities were studied in acetonitrile (Scheme 3, Table 1, Entries 1–3 and 7–9). Stirring of the reaction mixtures at room temperature led to 10β-fluorinated derivatives (**9** and **17**) solely, independent of the orientation of the angular methyl group (Table 1, Entries 1 and 7). Thus, 10β-fluoroestra-1,4-dien-3-ones (**9** and **17**) were formed in stereo- and chemoselective manners. Aromatic electrophilic substitutions at the ortho-positions (at C-2 or at C-4) did not occur. When fluorinations were carried out at 80 °C in acetonitrile, the same 10-fluoro products (**9** or **17**) were formed, but the reaction was complete within 1 h (Table 1, Entries 2 and 8). Our results are not consistent with those obtained for monosubstituted phenols [13], but they show good correspondence with chemoselectivities obtained earlier for fluorinations of estrone derivatives with Selectfluor [14,15,16,17]. In methanol, however, ortho-fluorinations also occurred (15–16%: **15**:**16** =1:0.9 or **18**:**19** =1:1.5) at both reaction temperatures (Table 1, Entries 4,5,10 and 11).

**Scheme 4:**
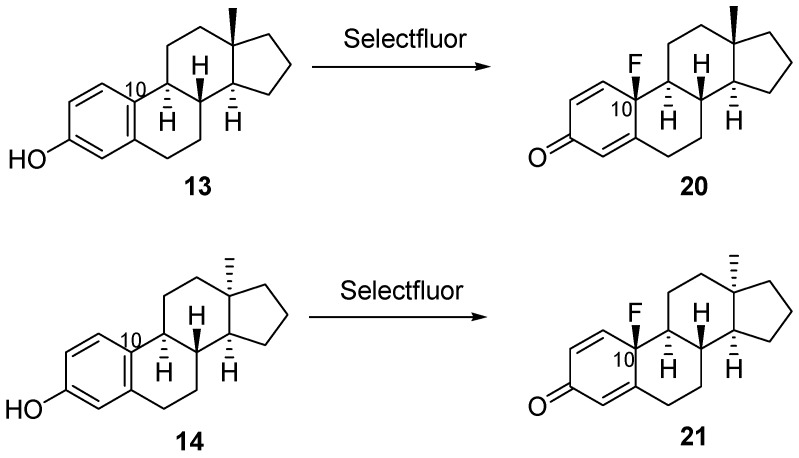
Fluorinations of 17-deoxyestrone (**13**) and 17-deoxy-13α-estrone (**14**) with Selectfluor (**2**).

In order to investigate the influence of minor structural modifications of the steroidal scaffold on the outcome of fluorinations, not only the 17-oxo compound, but also their 17-deoxy counterparts (**13** and **14**) were subjected to fluorinations in acetonitrile using **2** as the reagent (Scheme 4, Table 1, Entries 13, 14). As expected, the reactions proceeded chemo- and stereoselectively, resulting in 10β-fluoro-17-deoxy-estra-1,4-dien-3-ones (**20** and **21**) in both the 13β- and 13α-estrone series. It can be stated that the presence or lack of the 17-oxo function does not affect the outcome of these fluorination reactions.

**Scheme 5:**
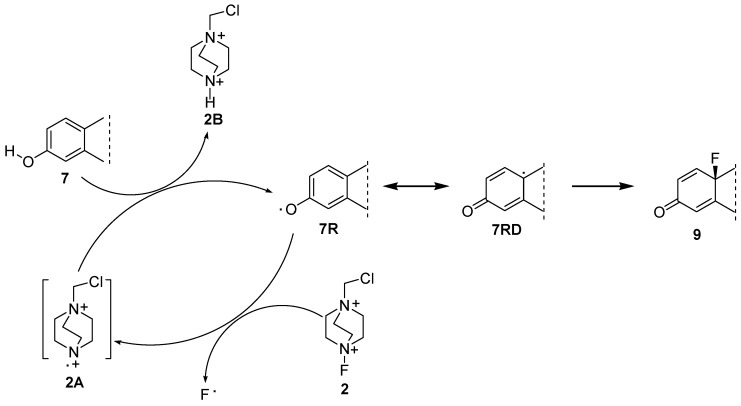
Proposed mechanism of fluorination of estrone (**7**) with Selectfluor (**2**) in acetonitrile or methanol.

According to the literature, the reaction mechanisms of fluorination reactions with Selectfluor (**2**) and other N–F fluorinating agents might be highly sensitive to the applied reaction conditions [21,22,23]. Different results have been observed with the same radical probe in different solvents. However, the ability of Selectfluor for homolytic cleavage of its N–F bond has recently been proved by Zhang et al., who detected the adduct of TEMPO (2,2,6,6-tetramethyl-1-piperidinyloxy) and the fluorine radical by LC-MS and F-NMR [24]. Based on these literature results, we investigated the mechanism of fluorination in acetonitrile and methanol by adding a radical scavenger to the reaction mixture (Scheme 5, Table 1, Entries 3, 6, 9, 12). Addition of 2 equiv. of TEMPO resulted in almost complete inhibition of fluorination, and only a trace of the desired 10-fluoro derivative (**9**) was formed. This indicates that fluorination presumably occurred via SET. Based on literature evidence [21,24] and our results, we assume that the homolytic cleavage of the N–F bond results in a fluorine radical and cationic nitrogen radical **2A**. The latter is protonated and intermediate **7R** is formed. Subsequent spin delocalization results in intermediate **7RD**. The driving force of this delocalization is the formation of a more stable tertiary radical. The attack of the fluorine radical on C-10 results in the desired 10-fluoro derivative **9**.

**Scheme 6:**
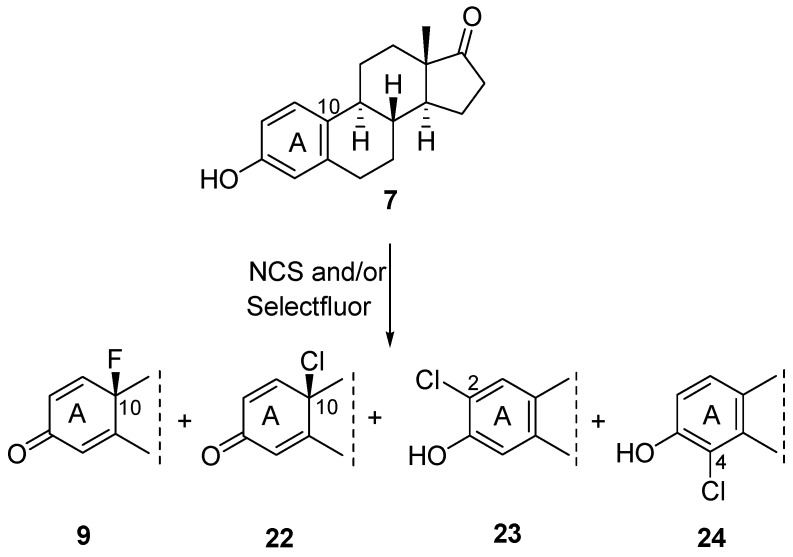
Reaction of estrone (**7**) with Selectfluor (**2**) and/or NCS.

One substrate, namely 13β-estrone **7**, was chosen for further derivatization. Chlorination of compound **7** was performed using N-chlorosuccinimide (NCS) as a reagent in acetonitrile and a catalytic amount of trifluoroacetic acid (TFA) (Scheme 6). Stirring the reaction mixture at rt for 24 h resulted in 2- and 4-chloro derivatives with retained aromaticity of ring A (**23** and **24**; Table 2, Entry 1). Heating the reaction mixture at 80 °C afforded the same product mixture with a much shorter reaction time of only 1 h (Table 2, Entry 2). Exchanging acetonitrile for TFA, ortho-chlorination was suppressed and 10-chloro dienone **22** became the main product (Table 2, Entries 3,4). The outcome of the reaction could not be influenced by adding TEMPO to the reaction mixture (Table 2, Entry 5). Based on the above-mentioned interesting results, model compound **7** was subjected to fluorination with Selectfluor, but acetonitrile used formerly was exchanged for TFA as the solvent. Fluorination occurred solely at C-10 (Table 2, Entries 6, 7). The formation of compound **9** could not be avoided by adding TEMPO to the reaction mixture (Table 2, Entry 8). In further experiments, the two halogenating agents (Selectfluor and NCS) were used together. In acetonitrile, fluorination occurred at C-10 together with ortho-chlorinated products **23** and **24** (Table 2, Entry 9). This ratio was retained by heating the reaction mixture at 80 °C for 1 h, but the starting compound was consumed earlier (Table 2, Entry 10). The two halogenating agents were also simultaneously used in TFA. In this process, the 10-fluoro and 10-chloro compounds (**9** and **22**) appeared to be the main products (Table 2, Entry 11). Heating shortened the reaction time, but it did not affect the product ratio (Table 2, Entry 12). TEMPO did not affect the outcome of this reaction either (Table 2, Entry 13).

The structures of the newly synthesized compounds (**17**, **20**, and **21**) were established through ^1^H- and ^13^C-NMR measurements. The configuration of the newly formed chiral center at C-10 in the 13α-epimer (**17**) was deduced from the comparison of the ^1^H-NMR spectra of the two 10-fluoro-13-epimeric compounds (**9** [16] and **17**). The multiplets of 1-H appeared with similar shapes and coupling constants in the two spectra, suggesting the same configuration (10β-F) of the new chiral center. It can be stated that owing to the long distance of the angular methyl group from ring A, the configuration of C-13 does not influence that of C-10.

### 2.2. Aromatase Inhibition Studies

Literature reveals that type I inhibitors for aromatase might be designed not only based on the substrate of the enzyme, but also on its product estrone (**7**) [1]. It was published that 2-halogenated (with F, Cl, and Br) estrone derivatives display high binding affinity to the enzyme with K_i_ values in the submicromolar or micromolar range [25]. The 17-Oxo analogs seemed to be more potent than the corresponding 17β-hydroxy compounds. It was stated that the presence of the 17-carbonyl function is necessary in the binding of estrogens to the active site of the aromatase enzyme [7,26,27,28]. The 4-halogenated derivatives proved to be less potent than their 2-substituted counterparts. We reported recently that 2-, 4- or 2,4-*bis*-halogenated (Cl, Br, I) 13α-estrones and their 17-deoxy derivatives possess weak aromatase inhibitory action [29]. It was demonstrated that the conformational differences of 13-epimeric estrones **7** and **12** resulting from the inversion of configuration at C-13 led to different binding affinities of 13-epimers to the aromatase. The inhibitory data obtained earlier for ring A halogenated 13β- and 13α-estrones indicate that the nature of the C-17 functional group, the conformation of the sterane core, and the substitution pattern of ring A might significantly influence the inhibitory behavior. 

**Figure 2:**
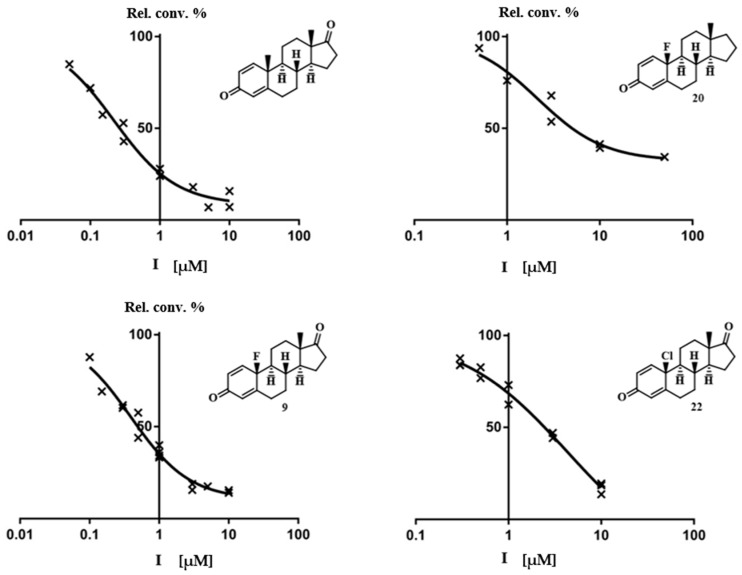
The concentration-dependent inhibitory action of the three potent compounds (**9**, **20** and **22**) and that of the reference compound androst-1,4-diene-3,17-dione.

Here, we expected that the binding affinity of estrane-based potential inhibitors might be improved by transforming the aromatic ring A into the 1,4-dien-3-one moiety. This structural element relates to that of the substrate of the enzyme. There exist numerous literature reports on the development of substrate-like aromatase inhibitors bearing the androsta-1,4-dien-3-one structure [1]. However, only a few estra-1,4-dien-3-ones have been evaluated for their inhibitory properties [30,31,32,33]. Our idea was to develop type I potential aromatase inhibitors, which possess the key structural elements, such as the 1,4-dien-3-one in ring A and the 17-oxo function, but instead of the C-19 methyl group, a promising fluorine substituent. We expected that the small, but highly electronegative fluorine will markedly improve the binding properties of the compounds. These structural modifications on the 13α-estrane core could result in compounds acting selectively without estrogenic behavior. The investigation of in vitro aromatase inhibitory action of test and reference compounds was performed by a radiosubstrate incubation method established previously [29]. Our experiments reveal that the 13β-epimer (**9**) was highly potent with an IC_50_ value in the submicromolar range (Table 3). This compound exerted similar potency to reference compounds androst-4-ene-3,17-dione and androst-1,4-diene-3,17-dione. Unfortunately, the 13α-epimer (**17**) displayed only a very weak inhibitory action. Concerning the 17-deoxy compounds (**20** and **21**), the same tendency was observed, namely that only the 13β derivative (**20**) inhibited the aromatase enzyme. The difference of one order of magnitude in the IC_50_ values of 17-oxo (**9**) and its 17-deoxy counterpart (**20**) indicates that the presence of a 17-keto function is advantageous in binding of the inhibitor. The 13α-epimer of the 17-deoxy derivative (**21**) displayed similar affinity to the enzyme to that of its 17-oxo (**17**) counterpart. The 10-chloro-13β-derivative (**22**) proved to be a potent inhibitor with an IC_50_ value in the low micromolar range, however literature reveals estrogenic activity for compound **22** comparable to that of estrone [34]. Figure 2 shows the concentration-dependent inhibitory action of the potent compounds (**9**, **20** and **22**) and that of the reference compound androst-1,4-diene-3,17-dione. The results obtained for 10-fluoro- and 10-chloro-13β compounds (**9** and **22**) suggest that introduction of a more electronegative but smaller halogen onto C-10 is more advantageous. 

### 2.3. Molecular Docking Studies

Having obtained an atomic level explanation concerning biological activity, computational simulations were carried on for all 5 ligands (**9**, **17**, **20**, **21**, and **22**) and the original androst-1,4-diene-3,17-dione molecule. Docking studies were performed by the Glide program [35,36,37], where the concerned receptor model was based on X-ray crystal structure selected from the PDB database (pdb code:3S79, [38]). The accuracy of the chosen protocol was verified by a redocking calculation, where a ligand from the X-ray complex was taken away and redocked into the original binding pocket. The XP docking protocol could reproduce the original binding pose with 0.1893 Å RMSD accuracy, but according to the Glide-score value, it was ranked in second position. Further information about the fitted X-ray and redocked ligand structures is presented in the supporting information (see Figure S1). Considering the Emodel scoring values, we had proper binding geometry and the original crystal position was ranked in the top position. This is in line with the theoretical background of the two scoring functions [37], as the Glide score was developed for large and diverse molecules set to maximize separation of compounds with strong or weak binding affinity. On the other hand, the Emodel value was developed for comparing analogous conformers, and much less for comparing chemically distinct species. Taking into account that our ligand set consists of very similar molecules, ligand poses with the best Emodel values were selected as docking results in each case and the corresponding scoring values are summarized in Table 4.

We can see that the Glide score could not separate characteristically active and inactive compounds, but the 13-epimer pairs (**9** vs. **17** and **20** vs. **21**) were always distinguished with proper binding preference order. Concerning the Emodel scores, the binding preference provided suitable separation of biologically active and inactive molecules as active molecules had better, i.e., more negative, scoring values. We would like to note that Androst-1,4-diene-3,17-done was also docked for reference reason, and it had −81.343 and −5.114 Emodel and Glide score values, respectively. This is in line with experiments, since this compound showed good biological activity (see Table 3).

**Figure 3:**
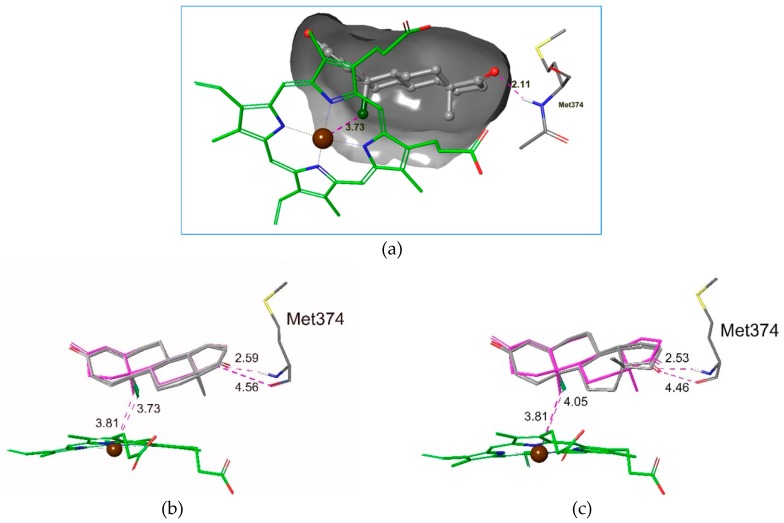
(**a**) The binding pose of ligand **22** with the best Emodel score. Distances are presented in Å. (**b**) The best binding pose of the biologically active ligands (**9**, **20**, and **22**) with the original X-ray ligand (molecule with purple line) pose. (**c**) The best binding pose of the biologically inactive ligands (**17** and **21**) with the original X-ray ligand (molecule with purple line) pose.

Focusing now on the binding geometry of the ligands, we presented the docking pose of ligand **22** with the best Emodel score in Figure 3a. It represents clearly the possible interaction with the heme group with the Fe as well as the H-bond between ligand **22** and the Met374 residue. Other parts of the ligands fitted into the binding pocket did not provide any characteristic interactions (e.g., H-bond or π–π stacking) with the surrounding amino acids. Additionally, the poses of biologically active and inactive ligands are also presented in Figure 3b,c, respectively, where the original ligand androstenedione geometry in the X-ray structure was also represented by purple wire. It demonstrated clearly that in the non-active cases (compounds **17** and **21**) the backbone had notable distortion, while biologically active molecules (**9**, **20** and **22**) provided almost exactly the same sterane skeleton arrangement when compared to the original X-ray geometry. The distortion of the sterane skeleton in the non-active cases (compounds **17** and **21**) was the consequence of inversion of C-13, which had an effect on the conformation of ring D as well. This certainly weakened the interaction between the 17-oxo function of ligand **22** and the Met374 amino acid, which was pointed out previously in the literature to be an important interaction [38]. However, we believe that this interaction is not the only important factor in the binding efficiency of the ligand, as in the case of compound **20**, which was found to be biologically active with the 17-oxo function completely missing. Moreover, compound **20** performed better concerning both biological activity and chemical binding than compound **17**, which bears the 17-oxo group.

It is worth mentioning that the docking calculations of 17-deoxy compound **21** provided a geometry where the orientation of the sterane skeleton was turned around, and the oxygen on ring A tried to play the role of the ring D oxygen in the interaction with the Met379. In this case, however, neither the Glide score nor the Emodel score indicated strong interaction.

From a theoretical point of view, these results put into focus two, not necessarily exclusive, explanations. On the one hand, there should be an interaction between the heme and the methyl groups and/or halogen groups in positions 13 and 10. This interaction, however, is weakened by the inversion at C-13. On the other hand, the inversion changed the hydrophobic interaction of the ligand in the binding pocket as well, which could also lead to a weaker ligand binding. Moreover, these effects obviously overrule the loss of the interaction with the Met379 residue. This became clear when we compared the scoring of ligand **20** to the results of ligand **17**.

## 3. Materials and Methods

### 3.1. Chemical Synthesis

#### 3.1.1. General

Melting points (Mp) were determined with a Kofler hot-stage apparatus and are uncorrected. Elemental analyses were performed with a Perkin-Elmer CHN analyzer model 2400. Thin-layer chromatography: silica gel 60 F_254_; layer thickness 0.2 mm (Merck, Darmstadt, Germany); eluents: a: 40% ethyl acetate/60% hexanes, b: 25% ethyl acetate/75% hexanes, detection with I_2_ or UV (365 nm) after spraying with 5 % phosphomolybdic acid in 50 % aqueous phosphoric acid and heating at 100–120 °C for 10 min. Flash chromatography: silica gel 60, 40–63 μm (Merck). ^1^H-NMR spectra were recorded in DMSO-*d*_6_, CDCl_3_ solution with a Bruker DRX-500 instrument at 500 MHz, with Me_4_Si as internal standard. ^13^C-NMR spectra were recorded with the same instrument at 125 MHz under the same conditions. Mass spectrometry: Full scan mass spectra of the compounds were acquired in the range of 50 to 1000 *m*/*z* with a Finnigan TSQ-7000 triple quadrupole mass spectrometer (Finnigan-MAT, San Jose, CA, USA) equipped with a equipped with a Finnigan electrospray ionization source. Analyses were performed in positive ion mode using flow injection mass spectrometry with a mobile phase of 50 % aqueous acetonitrile containing 0.1 *v*/*v* % formic acid. The flow rate was 0.3 mL/min. Five µl aliquot of the samples were loaded into the flow. The ESI capillary was adjusted to 4.5 kV and N_2_ was used as a nebulizer gas. 

#### 3.1.2. Synthesis of 10β-Fluoroestra-1,4-dien-3-one (**9**) or 10β-Fluoro-13α-estra-1,4-dien-3-one (**17**) in acetonitrile

Estrone (**7**) (135 mg, 0.5 mmol) or 13α-estrone (**12**) (135 mg, 0.5 mmol) was dissolved in acetonitrile (5 mL) and Selectfluor (**2**) (195 mg, 0.55 mmol) was added. The mixture was stirred at rt for 24 h or at 80 °C for 1 h, the solvent was then evaporated off, and the crude product (**9** or **17**) was purified by flash chromatography with 2% ethyl acetate/98% dichloromethane as eluent.

Compound **9** was obtained as a white solid (137 mg, 95% or 140 mg, 97%, Mp.: 104–102 °C, R_f_ = 0.42^a^). Compound **9** is identical with compound described in the literature [15]. ^1^H-NMR (DMSO-*d*_6_) δ ppm 0.86 (s, 3H, 18-H_3_); 2.41 and 2.53 (2 × m, 2 × 1H, 6-H_2_); 6.10 (s, 1H, 4-H); 6.22 (d, 1H, *J* = 10.2 Hz, 2-H); 7.27 (dd, 1H, *J* = 10.2 Hz, *J* = 7.4 Hz, 1-H).

Compound **17** was obtained as a white solid (140 mg, 97% or 141 mg, 98%, Mp.: 142–144 °C, R_f_ = 0.23^b^). Anal. Calcd. for C_18_H_21_FO_2_: C, 74.97; H, 7.34. Found: C, 74.85; H, 7.39. ^1^H-NMR (CDCl_3_) δ ppm 0.99 (s, 3H, 18-H_3_); 1.14–2.68 (15H); 6.04 (s, 1H, 4-H); 6.22 (d, 1H, *J*=10.2 Hz, 2-H); 7.06 (dd, 1H, *J* = 10.2 Hz, *J* = 7.7 Hz, 1-H). ^13^C-NMR (CDCl_3_) δ ppm 21.6; 23.6; 24.9 (C-18); 31.1; 31.5; 33.4; 34.0; 37.4; 49.1; 49.8 (C-13); 51.7 (d, *J* = 24.0 Hz, C-9); 88.9 (d, *J* = 167.9 Hz, C-10); 123.7 (d, *J* = 5.0 Hz, C-4); 129.7 (d, *J* = 8.7 Hz, C-2); 144.7 (d, *J* = 23.8 Hz, C-1); 159.8 (d, *J* = 18.9 Hz, C-5); 184.8 (C-3); 220.7 (C-17). MS *m*/*z* (%): 289 (100, [M + H]^+^).

#### 3.1.3. Synthesis of 10β-Fluoroestra-1,4-dien-3-one (**9**) or 10β-Fluoro-13α-estra-1,4-dien-3-one (**17**) in methanol

Estrone (**7**) (135 mg, 0.5 mmol) or 13α-estrone (**12**) (135 mg, 0.5 mmol) was dissolved in methanol (5 mL) and Selectfluor (**2**) (195 mg, 0.55 mmol) was added. The mixture was stirred at rt for 24 h or at 80 °C for 1 h, the solvent was then evaporated off, and the crude product (**9** or **17**) was purified by flash chromatography with 2% ethyl acetate/98% dichloromethane as eluent.

Starting from compound **7**, first eluted the mixture of **15**:**16** = 1:1.5 and was obtained as an oil (23 mg, 16% or 22 mg, 15%). Then eluted compound **9** and was obtained as a white solid (110 mg, 76% or 112 mg, 78%). Compounds **15** and **16** have not been separated. The relevant signals selected from the ^1^H-NMR spectrum of the mixture for compound **16** (DMSO-*d*_6_) δ ppm: 0.82 (s, 3H, 18-H_3_); 6.71 (t, 1H, *J* = 8.8 Hz, 2-H); 6.88 (d, 1H, *J* = 8.8 Hz, 1-H); 9.43 (s, 1H, OH). The relevant signals selected from the ^1^H-NMR spectrum of the mixture for compound **15** (DMSO-*d*_6_) δ ppm: 0.82 (s, 3H, 18-H_3_); 6.61 (d, 1H, *J* = 9.3 Hz, 4-H); 6.97 (d, 1H, *J* = 13.2 Hz, 1-H); 9.47 (s, 1H, OH). Then eluted compound **9** and was obtained as a white solid. 

Starting from compound **12**, first eluted the mixture of **18**:**19** = 1:1.5 and was obtained as an oil (17 mg, 12% or 19 mg, 13%). Compounds **18** and **19** have not been separated. The relevant signals selected from the ^1^H-NMR spectrum of the mixture for compound **18** (DMSO-*d*_6_) δ ppm: 0.96 (s, 3H, 18-H_3_); 6.58 (d, 1H, *J* = 9.2 Hz, 4-H); 6.97 (d, 1H, *J* = 13.9 Hz, 1-H); 9.47 (s, 1H, OH). The relevant signals selected from the ^1^H-NMR spectrum of the mixture for compound **19** (DMSO-*d*_6_) δ ppm: 0.96 (s, 3H, 18-H_3_); 6.69 (t, 1H, *J* = 8.8 Hz, 2-H); 6.88 (d, 1H, *J* = 8.8 Hz, 1-H); 9.42 (s, 1H, OH). Then eluted compound **17** and was obtained as a white solid (102 mg, 71% or 105 mg, 73%). 

#### 3.1.4. General Procedure for the Fluorination with Selectfluor in Acetonitrile or in Methanol by Adding TEMPO

Estrone (**7**) (135 mg, 0.5 mmol) or 13α-estrone (**12**) (135 mg, 0.5 mmol) was dissolved in acetonitrile (5 mL) or in methanol (5 mL), Selectfluor (**2**) (195 mg, 0.55 mmol) and TEMPO (156 mg, 1.0 mmol) were added. The mixture was stirred at rt for 24 h, and the reaction was monitored by TLC^a^. The crude products were purified by flash chromatography with 2% ethyl acetate/98% dichloromethane as eluent. Compound **9** or **17** was obtained as a white solid only in traces (2–4%). 

#### 3.1.5. Synthesis of 10β-Fluoro-17-deoxyestra-1,4-dien-3-one (**20**) or 10β-Fluoro-17-deoxy-13α-estra-1,4-dien-3-one (**21**)

17-Deoxy-estrone (**13**) (128 mg, 0.50 mmol) or 17-deoxy-13α-estrone (**14**) (128 mg, 0.50 mmol) was dissolved in acetonitrile (5 mL) and Selectfluor (**2**) (195 mg, 0.55 mmol) was added. The mixture was stirred at rt for 24 h, the solvent was then evaporated off, and the crude product was purified by flash chromatography with 5% ethyl acetate/95% hexane.

Compound **20** was obtained as an oil (129 mg, 94%, R_f_ = 0.72^b^). Anal. Calcd. for C_18_H_23_FO: C, 78.80; H, 8.45. Found: C, 78.71; H, 8.37. ^1^H-NMR (CDCl_3_) δ ppm 0.80 (s, 3H, 18-H_3_); 2.41 and 2.67 (2 × m, 2 × 1H, 6-H_2_); 6.03 (s, 1H, 4-H); 6.23 (d, 1H, *J* = 9.8 Hz, 2-H); 7.10 (t, 1H, *J* = 9.8 Hz, 1-H). ^13^C-NMR (CDCl_3_) δ ppm 17.3 (C-18); 20.3; 23.1; 25.7; 32.0; 33.5; 36.1; 38.1; 40.1; 40.9 (C-13); 53.2; 54.5 (d, *J* = 24.8 Hz, C-9); 88.9 (d, *J* = 167.8 Hz, C-10); 123.4 (d, *J* = 4.6 Hz); 129.2 (d, *J* = 8.5 Hz); 145.7 (d, *J* = 14.5 Hz, C-1); 161.0 (d, *J* = 18.6 Hz, C-5); 185.3 (C-3). MS *m*/*z* (%): 275 (100, [M + H]^+^).

Compound **21** was obtained as an oil (126 mg, 92%, R_f_ = 0.67^b^). Anal. Calcd. for C_18_H_23_FO: C, 78.80; H, 8.45. Found: C, 78.73; H, 8.35. ^1^H-NMR (CDCl_3_) δ ppm 0.89 (s, 3H, 18-H_3_); 2.39 and 2.63 (2 × m, 2 × 1H, 6-H_2_); 6.02 (s, 1H, 4-H); 6.22 (d, 1H, *J* = 10.3 Hz, 2-H); 7.11 (dd, 1H, *J* = 10.3 Hz, *J* = 7.5 Hz, 1-H). ^13^C-NMR (CDCl_3_) δ ppm 21.0; 22.2; 28.7; 29.7 (C-18); 31.9; 33.6; 34.3; 34.8; 37.3; 41.6 (C-13); 51.9; 52.6 (d, *J* = 24.1 Hz, C-9); 88.9 (d, *J* = 161.1 Hz, C-10); 123.4 (d, *J* = 4.7 Hz, C-4); 129.5 (d, *J* = 8.6 Hz, C-2); 145.2 (d, *J* = 14.4 Hz, C-1); 160.8 (d, *J* = 19.2 Hz, C-5); 185.1 (C-3). MS *m*/*z* (%): 275 (100, [M + H]^+^).

#### 3.1.6. Fluorination of estrone (**7**) with Selectfluor in TFA

Estrone (**7**) (135 mg, 0.5 mmol) was dissolved in TFA (3 mL) and Selectfluor (**2**) (195 mg, 0.55 mmol) was added. The mixture was stirred at rt for 24 h or at 80 °C for 1 h, then cooled to rt, poured onto 100 mL water and extracted with diethyl ether. The organic phase was dried over anhydrous sodium sulfate, filtered and evaporated. The crude product was purified by flash chromatography with 2% ethyl acetate/98% dichloromethane as eluent. Compound **9** was obtained as a white solid (138 mg, 96% or 137 mg, 95%).

#### 3.1.7. Fluorination of Estrone (**7**) with Selectfluor in TFA by Adding TEMPO

Estrone (**7**) (135 mg, 0.5 mmol) was dissolved in TFA (3 mL), Selectfluor (**2**) (195 mg, 0.55 mmol) and TEMPO (156 mg, 1.0 mmol) were added. The mixture was stirred at rt for 24 h, then poured onto 100 mL water and extracted with diethyl ether. The organic phase was dried over anhydrous sodium sulfate, filtered and evaporated. The crude product was purified by flash chromatography with 2% ethyl acetate/98% dichloromethane as eluent. Compound **9** was obtained as a white solid (137 mg, 95%).

#### 3.1.8. Chlorination of Estrone (**7**) with NCS in Acetonitrile 

Estrone (**7**) (135 mg, 0.5 mmol) was dissolved in acetonitrile (5 mL), trifluoroacetic acid (0.005 mL) and NCS (74 mg, 0.55 mmol) were added. The mixture was stirred at rt for 24 h or at 80 °C for 1 h, the solvent was then evaporated off, and the crude product was purified by flash chromatography with 10% ethyl acetate/90% hexane. First eluted compound **24** (37 mg, 24%), which was obtained as a white solid. Mp.: 181–183 °C, [34]: 272–274 °C, R_f_: 0.46^a^. Continued elution yielded the mixture of compounds **24** (32 mg, 21%) and **23** (26 mg, 17%). Finally eluted **23** (20 mg, 13%), which was obtained as a white solid. Mp.: 203–205 °C, [35]: 262–264 °C, R_f_: 0.42^a^. Compounds **23** and **24** are identical with those described in [28]. 

#### 3.1.9. Chlorination of Estrone (**7**) with NCS in TFA

Estrone (**7**) (135 mg, 0.5 mmol) was dissolved in TFA (3 mL) and NCS (74 mg, 0.55 mmol) was added. The mixture was stirred at rt for 24 h or at 80 °C for 1 h, and then cooled to rt, poured onto 100 mL water and extracted with diethyl ether. The organic phase was dried over anhydrous sodium sulfate, filtered and evaporated. The crude product was purified by flash chromatography with 10% ethyl acetate/90% hexane. First eluted **24** (20 mg, 13%). Continued elution yielded the mixture of **24** (11 mg, 7%) and **23** (9 mg, 5.6%). Then eluted **23** (7 mg, 4.4%). Finally eluted **22** (84 mg, 55%).

Compound **22** was obtained as a white solid. ^1^H-NMR (CDCl_3_) δ ppm 0.98 (s, 3H, 18-H_3_); 6.09 (s, 1H, 4-H); 6.20 (d, 1H, *J* = 10.1 Hz, 2-H); 7.12 (d, 1H, *J* = 10.1 Hz, 1-H). ^13^C-NMR (CDCl_3_) δ ppm 13.7 (C-18); 21.9; 22.4; 30.6; 31.4; 32.0; 35.5; 35.6; 47.6 (C-13); 49.7; 53.2; 67.5 (C-10); 124.1; 126.9; 147.4 (C-1); 160.4 (C-5); 184.9 (C-3); 219.7 (C-17). Compound **22** is identical with compound described in the literature [34].

#### 3.1.10. Chlorination of estrone (**7**) with NCS in TFA by adding TEMPO

Estrone (**7**) (135 mg, 0.5 mmol) was dissolved in TFA (3 mL), NCS (74 mg, 0.55 mmol) and TEMPO (156 mg, 1.0 mmol) were added. The mixture was stirred at 80 °C for 1 h, and then cooled to rt, poured onto 100 mL water and extracted with diethyl ether. The organic phase was dried over anhydrous sodium sulfate, filtered and evaporated. The crude product was purified by flash chromatography with 10% ethyl acetate/90% hexane. First eluted **24** (21 mg, 14%). Continued elution yielded the mixture of **24** (11 mg, 7%) and **23** (10 mg, 7.0%). Then eluted **23** (6 mg, 4.0%). Finally eluted **22** (82 mg, 54%).

#### 3.1.11. Fluorination and Chlorination of Estrone (7) with Selectfluor and NCS in Acetonitrile 

Estrone (**7**) (135 mg, 0.5 mmol) was dissolved in acetonitrile (5 mL), Selectfluor (**2**) (195 mg, 0.55 mmol), NCS (74 mg, 0.55 mmol) and trifluoroacetic acid (0.005 mL) were added. The mixture was stirred at rt for 24 h or at 80 °C for 1 h, the solvent was then evaporated off, and the crude product was purified by flash chromatography with 10% ethyl acetate/90% hexane. First eluted **24** (15 mg, 9.8%). Continued elution yielded the mixture of **24** (14 mg, 9.2%) and **23** (10 mg, 6.6%). Then eluted **23** (12 mg, 8.4%). Finally eluted **9** (94 mg, 62%).

#### 3.1.12. Fluorination and Chlorination of Estrone (**7**) with Selectfluor and NCS in TFA 

Estrone (**7**) (135 mg, 0.5 mmol) was dissolved in TFA (3 mL), Selectfluor (**2**) (195 mg, 0.55 mmol) and NCS (74 mg, 0.55 mmol) were added. The mixture was stirred at 80 °C for 1 h, and then cooled to rt, poured onto 100 mL water and extracted with diethyl ether. The organic phase was dried over anhydrous sodium sulfate, filtered and evaporated. The crude product was purified by flash chromatography with 10% ethyl acetate/90% hexane. First eluted **24** (14 mg, 9.2%). Continued elution yielded the mixture of **24** (10 mg, 6.8%) and **23** (9 mg, 5.9%). Then eluted **23** (8 mg, 5.1%). Finally eluted separately **22** (40 mg, 26%) **9** (55 mg, 36%).

#### 3.1.13. Fluorination and Chlorination of Estrone (**7**) with Selectfluor and NCS in TFA by Adding TEMPO

Estrone (**7**) (135 mg, 0.5 mmol) was dissolved in TFA (3 mL), Selectfluor (**2**) (195 mg, 0.55 mmol), NCS (74 mg, 0.55 mmol) and TEMPO (156 mg, 1.0 mmol) were added. The mixture was stirred at 80 °C for 1 h, and then cooled to rt, poured onto 100 mL water and extracted with diethyl ether. The organic phase was dried over anhydrous sodium sulfate, filtered and evaporated. The crude product was purified by flash chromatography as it is described in Section 3.1.11.

### 3.2. Aromatase Inhibition

#### 3.2.1. General

[4(N)-^14^C]Testosterone was obtained from American Radiolabeled Chemicals, St. Louis, MO, USA). Chemicals and solvents of analytical grade purity were purchased from Sigma (St. Louis, MO, USA), from Fluka (Buchs, Switzerland) or from Merck (Darmstadt, Germany).

#### 3.2.2. Preparation of Enzyme Sources

Human term placentae were collected immediately after delivery and stored frozen at −80 °C. Tissue specimens were homogenized with an Ultra-Turrax in 0.1 M HEPES buffer solution (pH = 7.3) containing 1 mM EDTA and 1 mM dithiotreitol, and microsomas were obtained with fractionated centrifugation. Application of the human tissue was approved by the institutional Human Investigation Review Board. 

#### 3.2.3. Incubation Procedures

The microsoma suspension was incubated with 1.0 μM [4(N)-^14^C]testosterone substrate in the presence of 0.1 mM NADPH cofactor excess. Enzymatic incubations were carried out in the HEPES buffer medium at a final volume of 200 µL. The substrate was added to the incubate in 20 μL of a 25 *v*/*v*% propylene glycol in HEPES buffer solution, whereas test compounds were applied in 10 μL of dimethyl sulfoxide solution. Incubations were performed at 37 °C and lasted for 40 min. Enzymatic reaction was terminated by cooling and the addition of organic solvents of the subsequent extraction procedure. Control samples with no inhibitor and blank samples were incubated simultaneously. The incubation mixture was extracted with toluene, then the toluene phase was drained and washed with HEPES buffer. Aromatase products containing phenolic hydroxy group were extracted with 1.2 M sodium hydroxide solution from the toluene extract and radioactivity of the alkaline phase was measured in liquid scintillation counting.

#### 3.2.4. Inhibition Studies

In the general procedure, test compounds were applied at 10 μM final concentration in the incubate. Relative conversions compared to non-inhibited controls (100%) were determined. The assays were performed in triplicate, and the mean value and the standard deviation (SD) were calculated. IC_50_ values (the inhibitor concentration that decreases the enzyme activity to 50%) were determined for the most effective test compounds and for reference compounds. In these cases, conversions were measured at 10–15 different concentrations in the appropriate interval between 0.001–50 μM. IC_50_ results were calculated by using unweighted iterative least squares logistic curve fitting by means of the “absolute IC_50_ calculation“ function of the GraphPad Prism 4.0 software (GraphPad Software, Inc., San Diego, CA, USA).

### 3.3. Computational Simulations

Docking studies were performed with the Glide [33,34,35] program from the Schrodinger suits using the XP protocol. Docking grid generation was based on X-ray crystal structure as it was deposited into the Protein Database (pdb code: 3S79) and refined by the Protein Preparation Wizard. During the XP docking calculations, enhanced sampling was selected and the energy window for ring sampling was also increased to 100 kcal/mol and the number of final outputs per ligand was enlarged to 10. All figures were prepared with the Maestro program [35] which is the GUI part of the Schrödinger program package.

## 4. Conclusions

In conclusion, fluorinations of 13-epimeric estrones (**7** and **12**) and their 17-deoxy counterparts (**13** and **14**) with Selectfluor (**2**) in acetonitrile furnished exclusively the corresponding 10-fluoro derivatives (**9**, **17**, **20**, and **21**). Mechanistic studies suggest that reactions in acetonitrile occur via SET, while halogenations in TFA follow a different mechanism. The simultaneous application of the two halogenating agents (Selectfluor and NCS) in TFA results in 10-halogenated compounds **9** and **22** as the main products at a ratio of about 1.3:1.0. The results obtained from the aromatase assay suggest that in estrane-based aromatase inhibitors, the presence of the small, β-oriented halogen at C-10 and the 1,4-diene-3,17-dione moiety are advantageous and might lead to potent derivatives with submicromolar inhibitory potential. Docking calculations reinforced that besides the well-known Met374 H-bond connection, the stereocenter in the 13 position has an important role in the binding affinity. Our results might contribute not only to the research field of type I steroidal inhibitors of aromatase, but also to the development of biologically active compounds bearing substituted phenol moieties, by improving their biological potency via halogenations.

## Figures and Tables

**Table 1:** Fluorinations of estrone (**7**), 13α-estrone (**12**), 17-deoxyestrone (**13**) or 17-deoxy-13α-estrone (**14**) with Selectfluor (**2**).

Entry	Substrate	Solvent	Temperature	Reaction Time	Product	Yield (%)
1	**7**	acetonitrile	rt	24 h	**9**	95
2	**7**	acetonitrile	80 °C	1 h	**9**	97
3 ^a^	**7**	acetonitrile	rt	24 h	**9**	3
4	**7**	methanol	rt	24 h	**9** + (**15** + **16**) ^b^	76 + (16)
5	**7**	methanol	reflux	1 h	**9** + (**15** + **16**)	78 + (15)
6 ^a^	**7**	methanol	rt	24 h	**9**	2
7	**12**	acetonitrile	rt	24 h	**17**	97
8	**12**	acetonitrile	80 °C	1 h	**17**	98
9 ^a^	**12**	acetonitrile	rt	24 h	**17**	4
10	**12**	methanol	rt	24 h	**17** + (**18** + **19**) ^c^	71 + (12)
11	**12**	methanol	reflux	1 h	**17** + (**18** + **19**)	73 + (13)
12 ^a^	**12**	methanol	rt	24 h	**17**	94
13	**13**	acetonitrile	rt	24 h	**20**	94
14	**14**	acetonitrile	rt	24 h	**21**	92

^a^ 2 equiv. of 2,2,6,6-tetramethyl-1-piperidinyloxy (TEMPO); ^b^ Ratio: **15**:**16** = 1:0.9; ^c^ Ratio: **18**:**19** = 1:1.5.

**Table 2:** Effect of the reaction conditions on the fluorination and/or chlorination of compound **7**.

Entry	Substrate	NCS and or Selectfluor (1.1 equiv.)	Solvent	Temp.	Reaction Time	Yield Products 9 + 22 + 23 + 24 (%)
1 ^a^	**7**	NCS	acetonitrile	rt	24 h	0 + 0 + 30 + 45
2 ^a^	**7**	NCS	acetonitrile	80 °C	1 h	0 + 0 + 30 + 45
3	**7**	NCS	TFA	rt	24 h	0 + 55 + 10 + 20
4	**7**	NCS	TFA	80 °C	1 h	0 + 55 + 10 + 20
5 ^b^	**7**	NCS	TFA	80 °C	1 h	0 + 54 + 11 + 21
6	**7**	Selectfluor	TFA	rt	24 h	96 + 0 + 0 + 0
7	**7**	Selectfluor	TFA	80 °C	1 h	95 + 0 + 0 + 0
8 ^b^	**7**	Selectfluor	TFA	rt	24 h	95 + 0 + 0 + 0
9 ^a^	**7**	NCS, Selectfluor	acetonitrile	rt	24 h	62 + 0 + 15 + 20
10 ^a^	**7**	NCS, Selectfluor	acetonitrile	80 °C	1 h	62 + 0 + 15 + 20
11	**7**	NCS, Selectfluor	TFA	rt	24 h	36 + 26 + 11 + 16
12	**7**	NCS, Selectfluor	TFA	80 °C	1 h	36 + 26 + 11 + 16
13 ^b^	**7**	NCS, Selectfluor	TFA	80 °C	1 h	36 + 25 + 11 + 17

^a^ catalytic amount of TFA; ^b^ 2 equiv. of TEMPO.

**Table 3:** In vitro inhibition of aromatase activities by the test compounds and reference agents. Relative conversions (Rel. conv., control incubation with no inhibition is 100%) measured in the presence of 10 μM concentration of the compound tested. Mean ± SD, *n* = 3. IC_50_: inhibitor concentration decreasing the enzyme activity to 50%. SD: standard deviation.

Compound	Structure	IC_50_ ± SD (µM) or Rel. conv. ± SD (%)
**9**	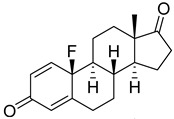	IC_50_ = 0.49 ± 0.07
**20**	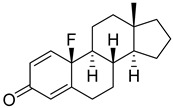	IC_50_ = 5.0 ± 2.4
**17**	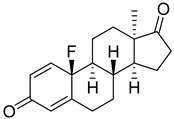	IC_50_ > 10 93 ± 11
**21**	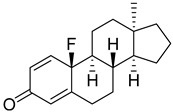	IC_50_ > 10 100 ± 6
**22**	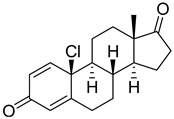	IC_50_ = 2.4 ± 0.4
Androst-4-ene-3,17-dione	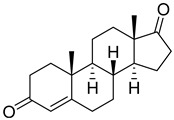	IC_50_ = 0.22 ± 0.2
Androst-1,4-diene-3,17-dione	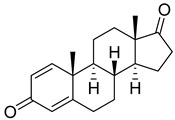	IC_50_ = 0.26 ± 0.06

**Table 4:** The scoring values for compounds **9**, **17**, and **20**–**22**.

Compound	Emodel Score	Glide Score
**22**	−80.929	−4.973
**9**	−79.415	−4.626
**20**	−72.055	−4.192
**21**	−64.406	−3.642
**17**	−61.448	−3.642

## Supplementary Materials

The following are available online at https://www.mdpi.com/1420-3049/24/9/1783/s1: Synthetic procedures, characterization data for the synthesized compounds, aromatase inhibition method and computational simulation methods.
