# Supplementary material for: Synthesis, Biological Evaluation and Docking Studies of 13-Epimeric 10-fluoro- and 10-Chloroestra-1,4-dien-3-ones as Potential Aromatase Inhibitors
Source: Molecules. 2019 May 8;24(9):1783. doi: 10.3390/molecules24091783 (PMC6540200; doi:10.3390/molecules24091783)
Supplement: Supplementary file 1 [file molecules-24-01783-s001.zip › molecules-489208-SI/Figure S1.pdf]

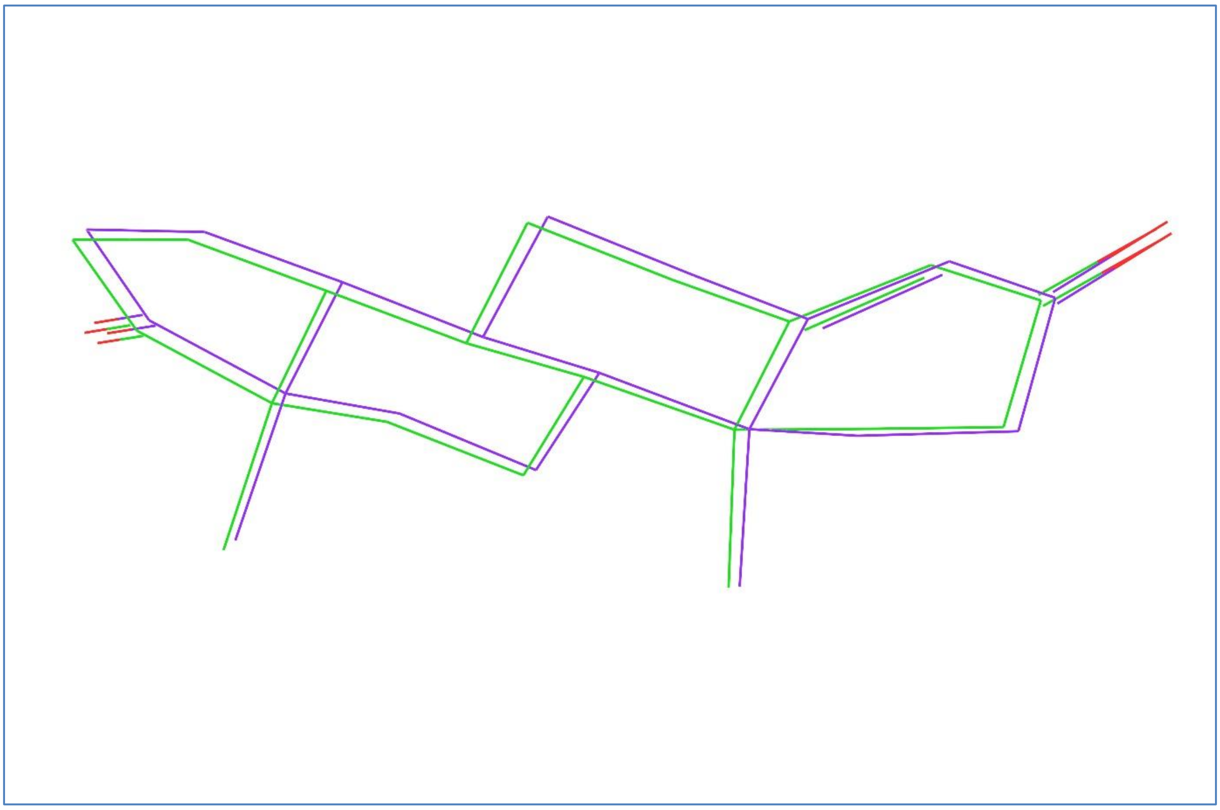

Figure S1 The superimposed ligand structures of the original X-ray position (purple line) and the redocked ligand with the best Emodel score (green and red lines). Only the heavy atoms are presented in the figure, and the RMSD between the two structures is 0.1893 Å.
