# Supplementary figures and images for: Synthesis, Biological Evaluation and Docking Studies of 13-Epimeric 10-fluoro- and 10-Chloroestra-1,4-dien-3-ones as Potential Aromatase Inhibitors
Source: Molecules. 2019 May 8;24(9):1783. doi: 10.3390/molecules24091783 (PMC6540200; doi:10.3390/molecules24091783)

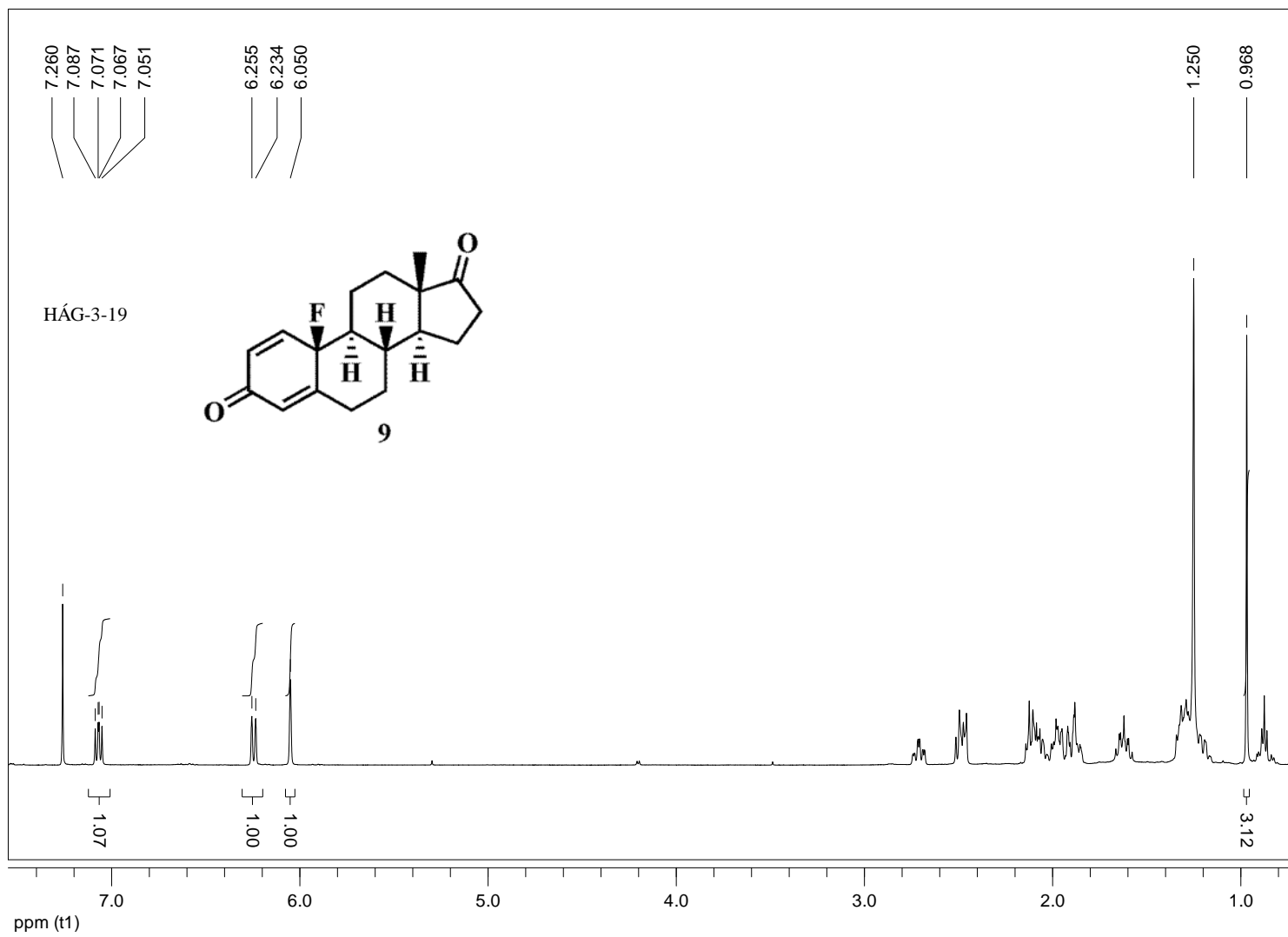

HÁG-3-19

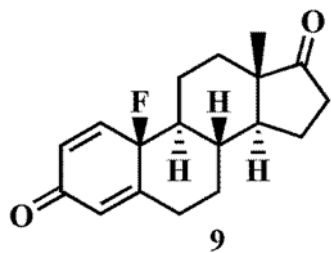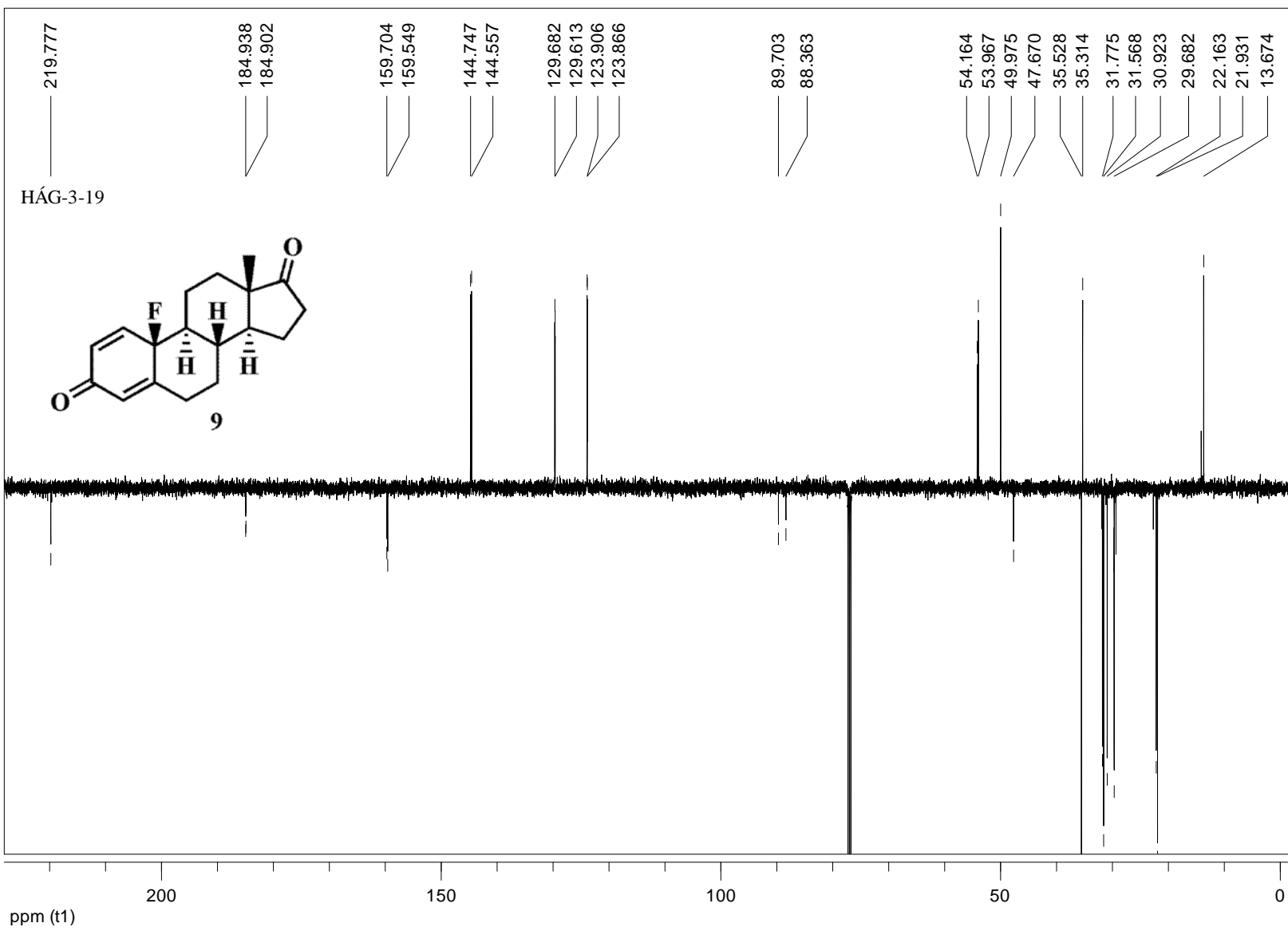

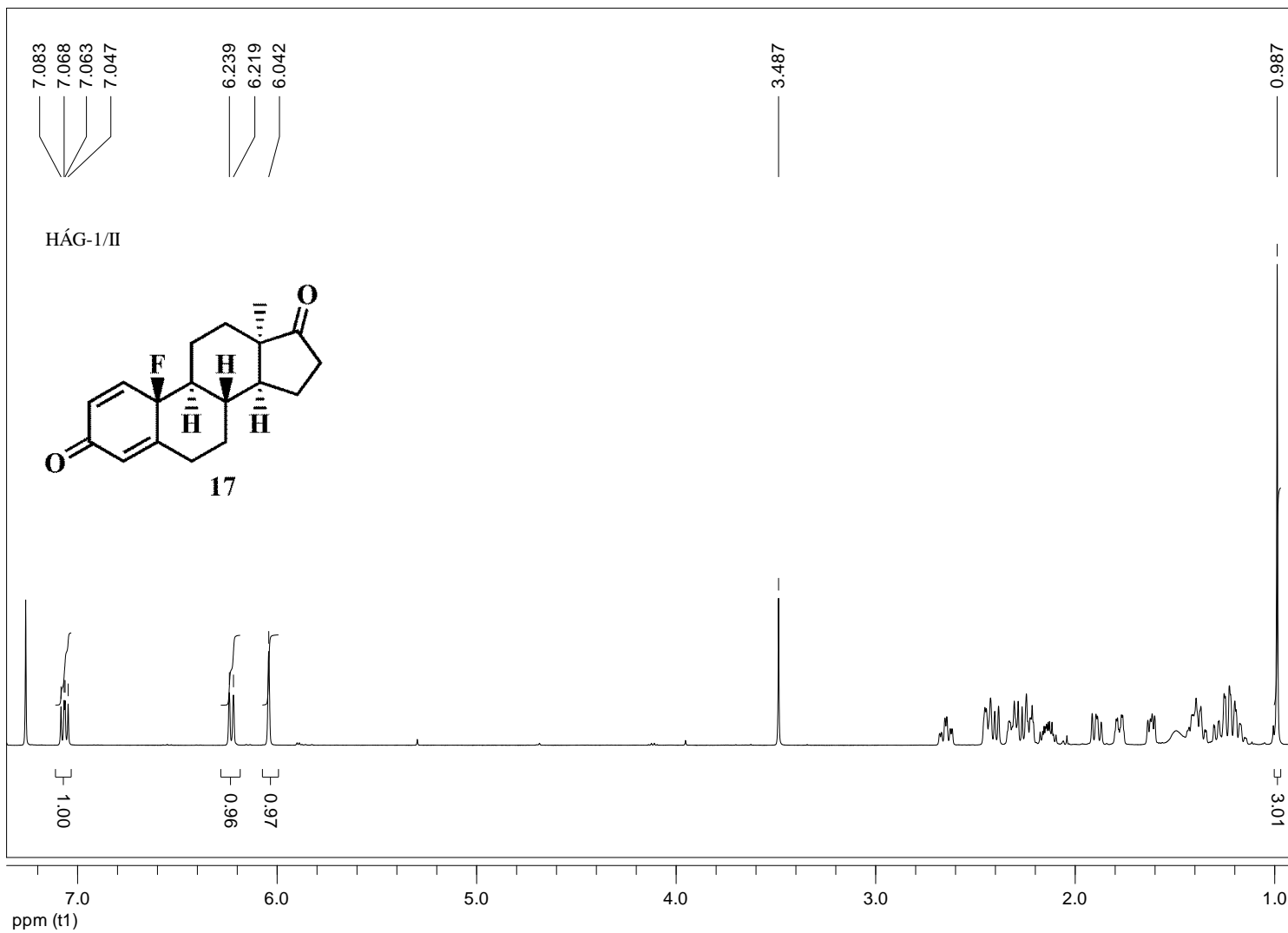

HÁG-1/II

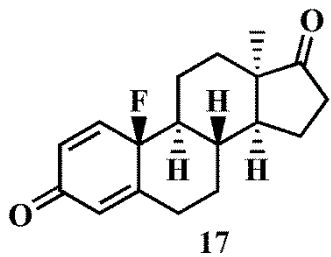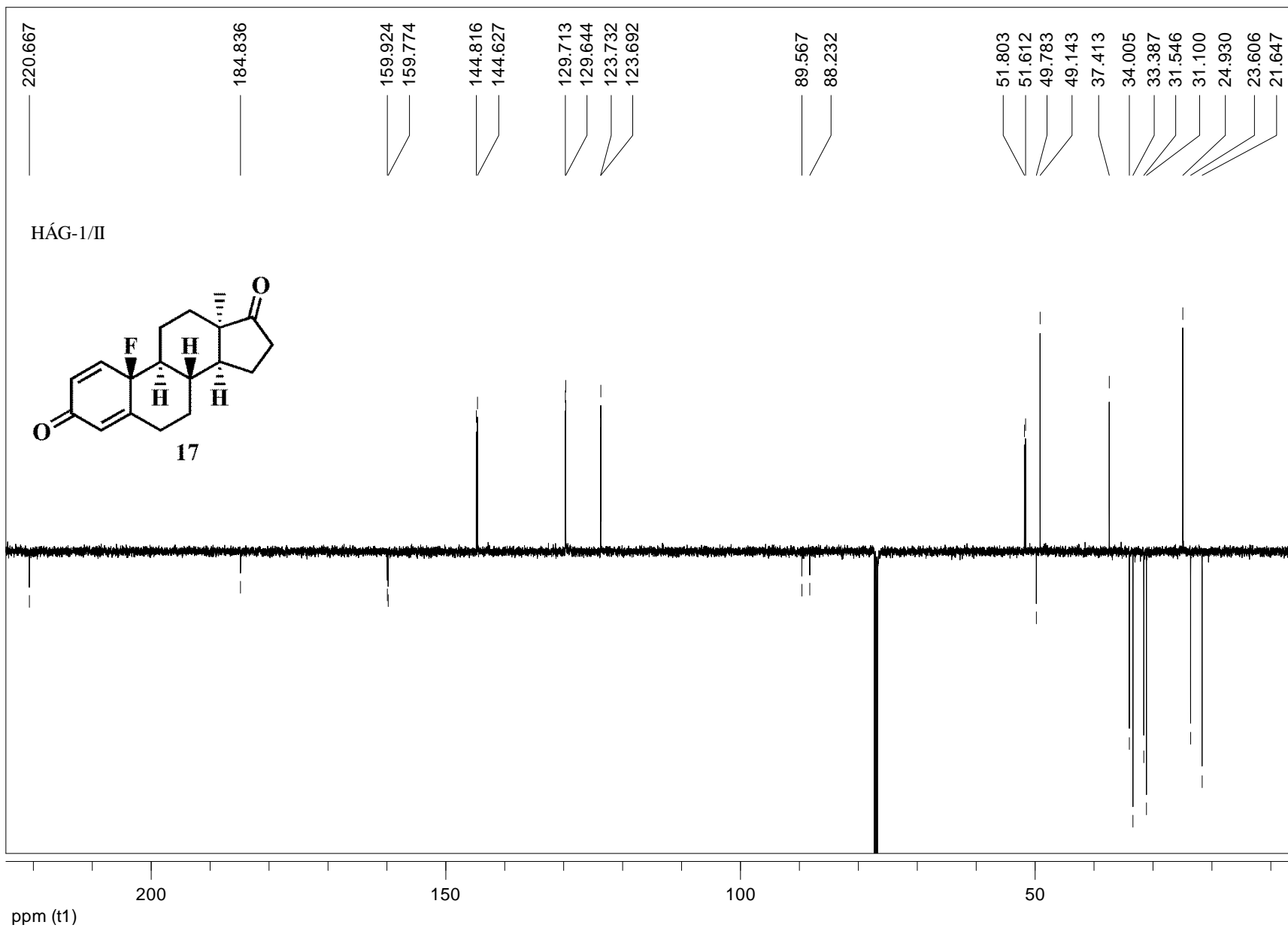

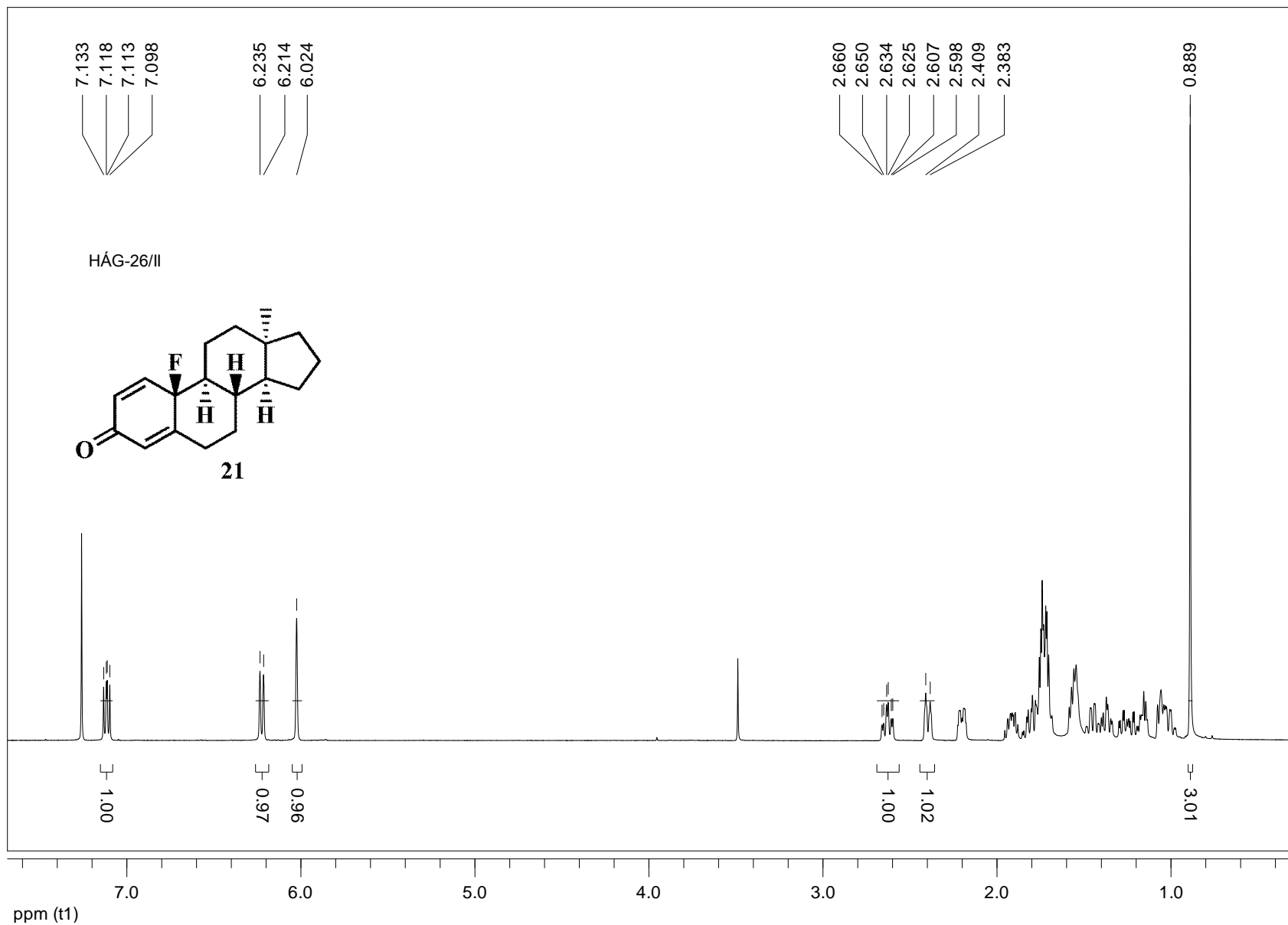

HÁG-26/II

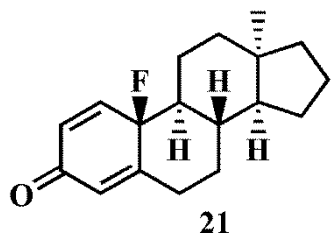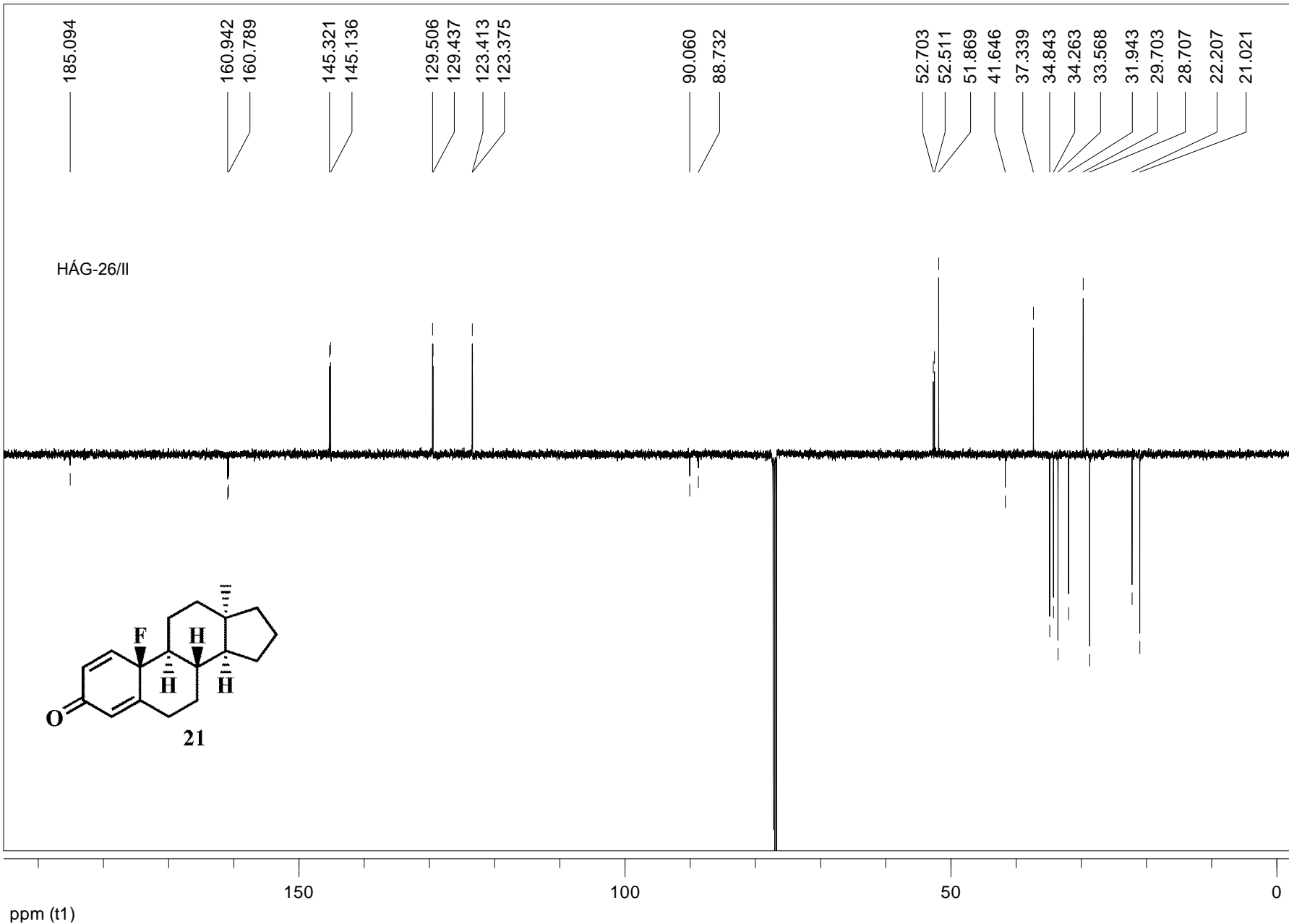

7.118  
7.100  
7.083

6.242  
6.223  
6.033

2.697  
2.673  
2.654  
2.429  
2.404

0.802

HÁG-31/4,5

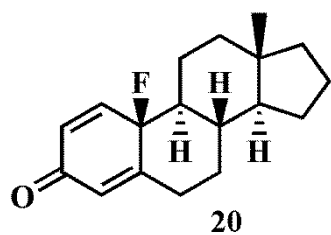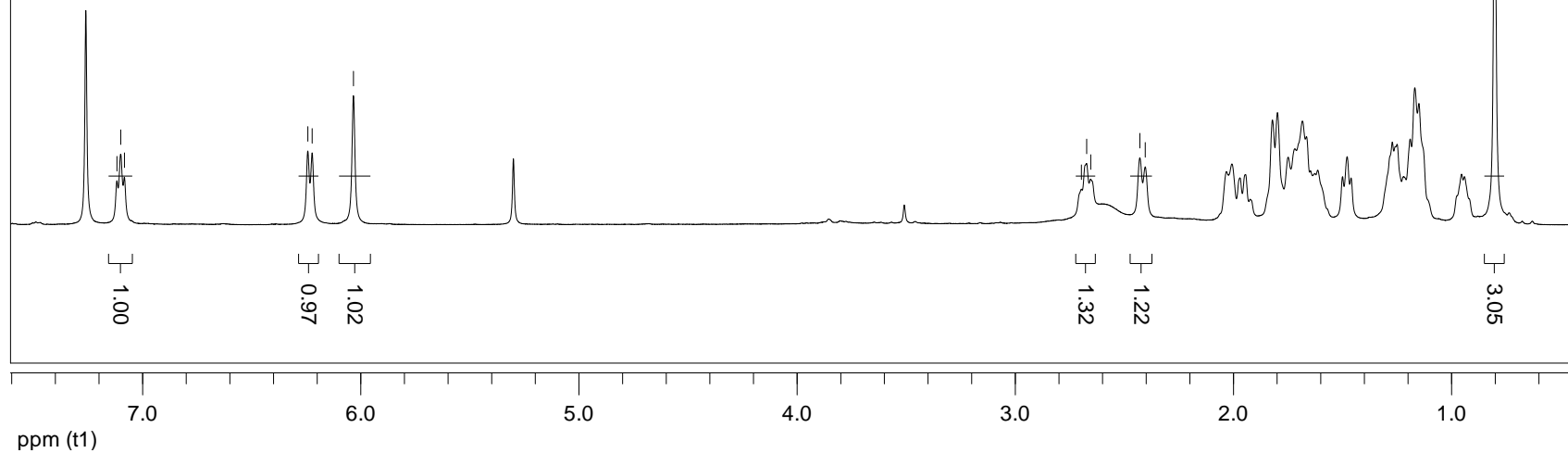

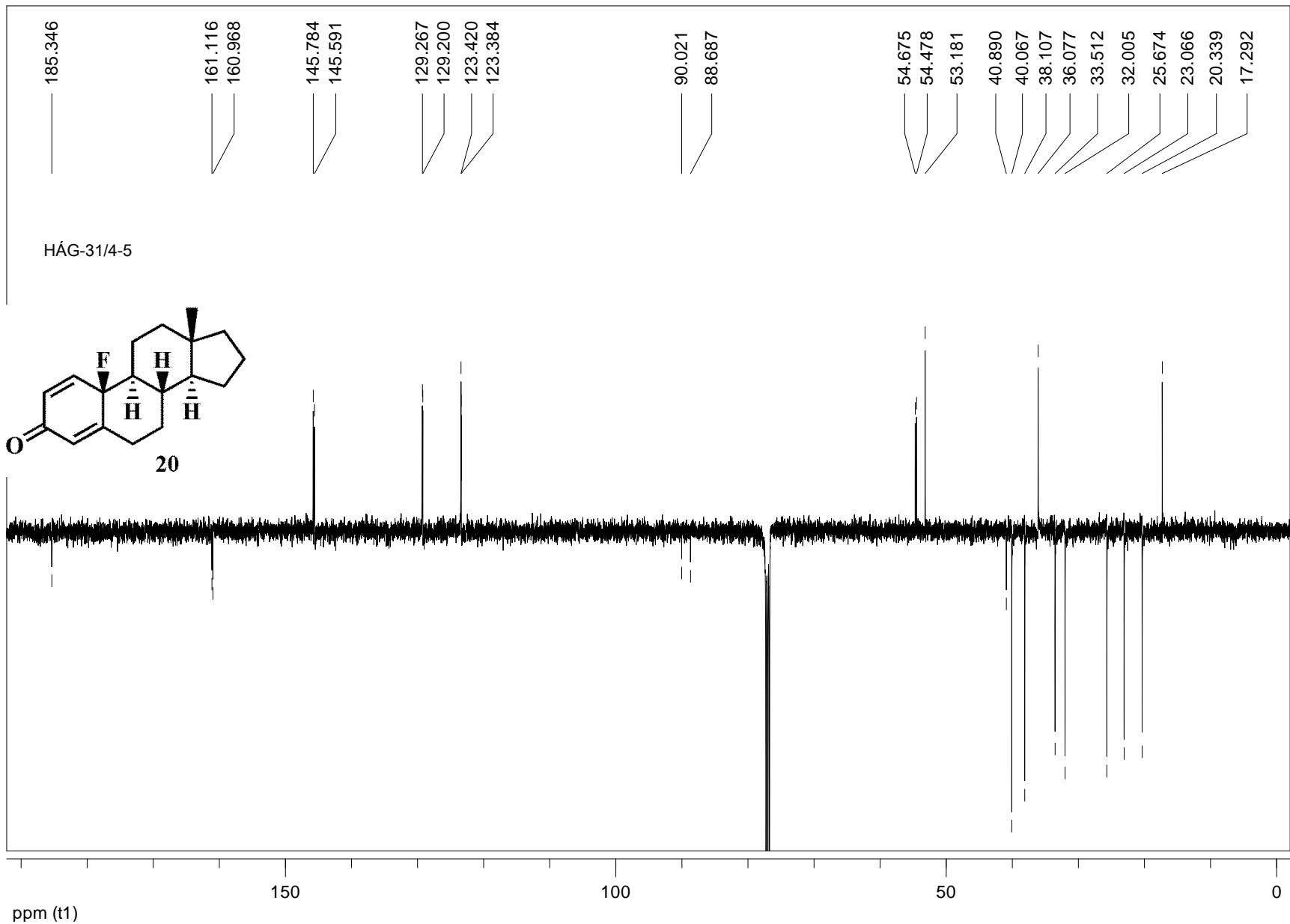

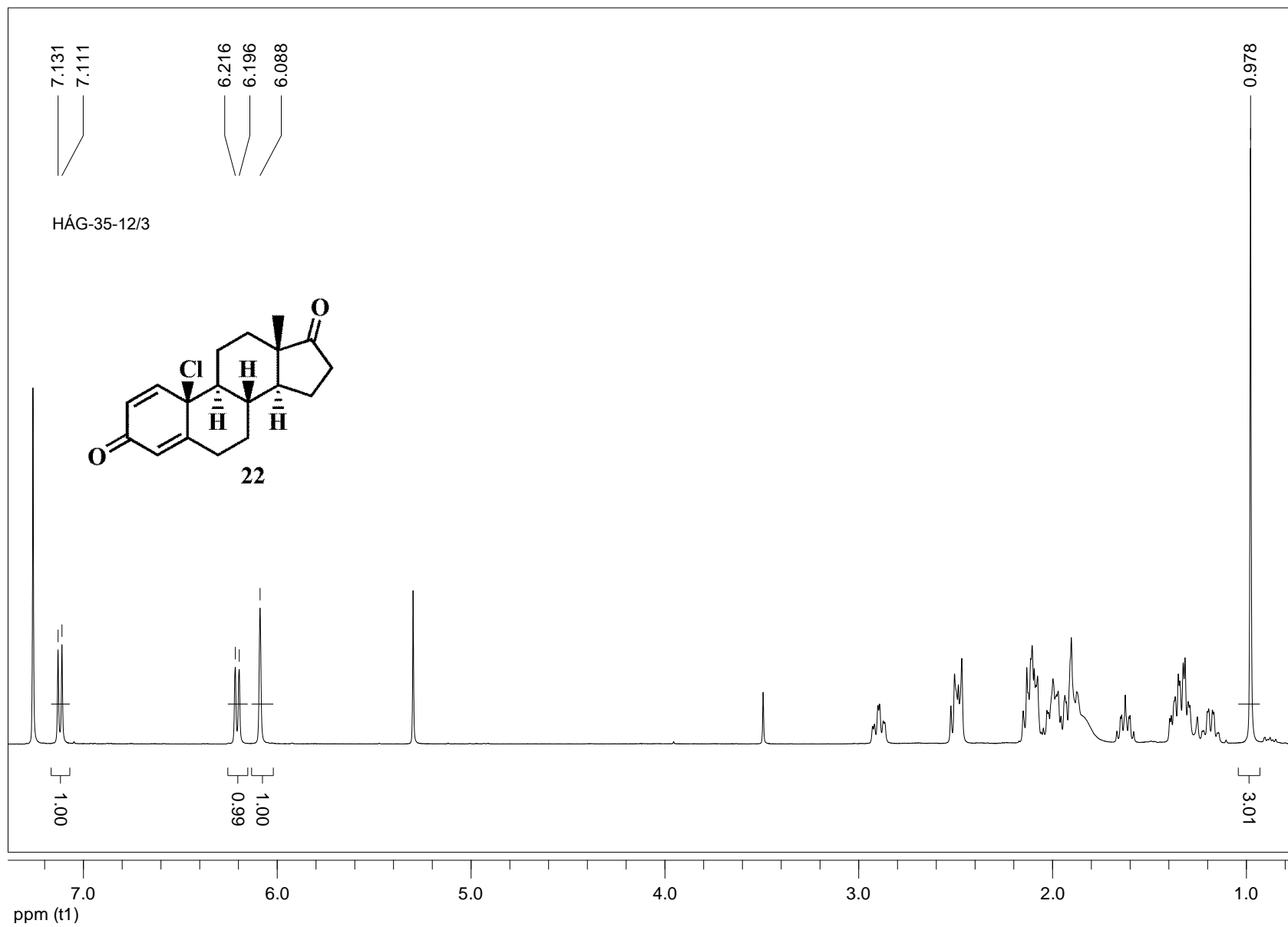

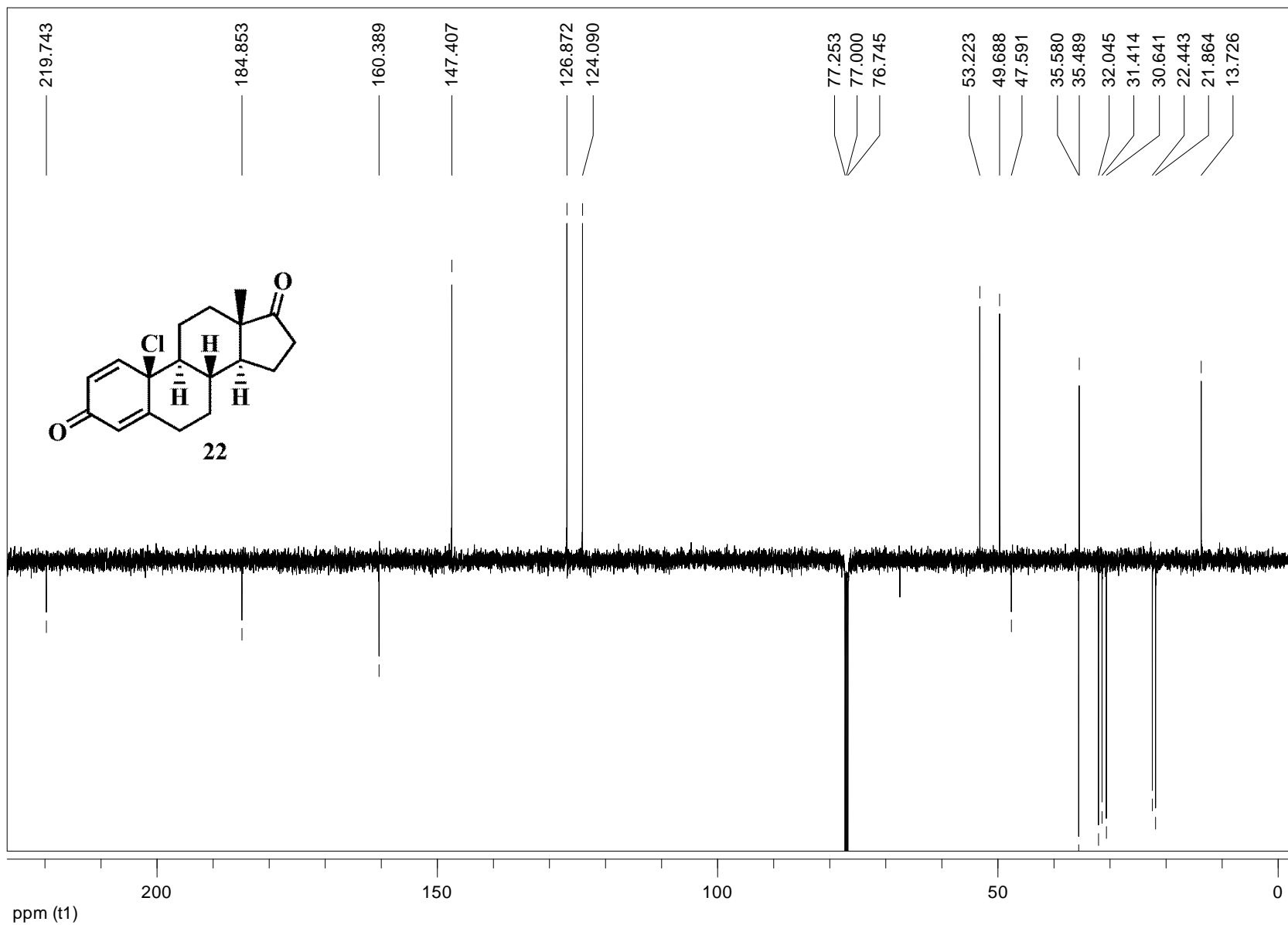

Supplement: Supplementary file 1 [file molecules-24-01783-s001.zip › molecules-489208-SI/Supplementary NMR spectra.pdf]
